# Digital nudging techniques for behaviour change in lifestyle medicine: a scoping review

**DOI:** 10.3389/fdgth.2026.1799205

**Published:** 2026-07-30

**Authors:** David Taniar, Emma King, Sam Manger, Karen Carlisle

**Affiliations:** 1 Faculty of Information Technology, Monash University, Clayton, VIC, Australia; 2 Victorian Heart Institute, Monash University, Clayton, VIC, Australia; 3 College of Medicine and Dentistry, James Cook University, Townsville, QLD, Australia

**Keywords:** digital nudging, emerging technology, health-related behaviour change, lifestyle medicine, nutrition, physical activity

## Abstract

Digital nudging has emerged as a promising strategy for promoting health-related behaviour change within the context of Lifestyle Medicine (LM). This scoping review systematically mapped the current evidence on digital nudging interventions across pillars of LM, including nutrition, physical activity (PA), mind-body health, sleep, and addiction. A total of 52 studies were included, covering a range of technologies, such as mobile apps, web-based platforms, augmented reality, chatbots, and voice-activated systems. These technologies served as platforms or mechanisms for delivering digital nudges rather than constituting distinct nudging categories. Through thematic analysis, digital nudging interventions were categorised into four types: (i) one-way nudging, (ii) nudging through system interactions, (iii) nudging via peer interaction, and (iv) self-nudging. Nutrition and physical activity were the most frequently targeted LM domains, accounting for more than three-quarters of the studies. The review highlights the potential of digital nudges to drive behaviour change, particularly when integrated with Artificial Intelligence (AI) or advanced technologies. However, gaps remain, including short-term study designs, limited diversity in participant populations, and underrepresentation of certain LM pillars. Future research should prioritise long-term studies with broader and more diverse populations. Enhanced interdisciplinary collaboration is crucial to advancing the design and implementation of effective digital nudging strategies in lifestyle medicine.

## 1 Introduction

Lifestyle medicine (LM) has been dubbed the future of chronic disease prevention, treatment, management, and reversal (1–3). Lifestyle medicine is an emerging discipline that uses behaviour change theory and coaching techniques to help patients develop lasting habits in key areas, such as healthy eating, physical activity, improved sleep, stress management, avoidance of harmful substances, and maintaining healthy relationships (4).

However, changing health-related behaviour is known to be difficult (5). Knowledge alone is rarely sufficient to change behaviour (6). Behaviour change, especially related to a healthy lifestyle, needs complex approaches. While understanding a condition and its management is crucial, it often does not lead to sustained behavioural changes without additional support mechanisms (7).

Nudging is a tool often used to motivate behaviour change. Nudge theory is a form of choice architecture that leverages indirect suggestions to influence behaviour (8, 9). Nudge generally requires minimal intervention, is not invasive, and is easy to avoid without significantly changing incentives or forbidding any options. Beyond commercial settings, nudging is increasingly used to create positive behaviour change, including health-related behaviour, such as changing the order of food displays to influence customers to purchase healthier food (8).

Digital technologies are playing an increasingly prominent role in promoting well-being. While they were not originally designed to drive behaviour change, these technologies have evolved into persuasive tools (10). Many of these persuasive technologies take the form of mobile apps, wearables, web applications, and virtual reality. The key question is how these technologies can employ nudging techniques to facilitate behaviour change, support the development of healthy habits, and prevent and manage chronic diseases.

Several reviews have addressed digital nudging and behaviour change (11–16). However, there is a gap in the literature reviewing digital nudging in the context of lifestyle interventions and lifestyle medicine. Therefore, the primary aim of this scoping review is to understand the strengths and limitations of each digital nudging technique for behaviour change in lifestyle medicine-related domains and conditions.

This question posts three main aspects: (1) behaviour change nudging techniques, (2) digital technology, and (3) lifestyle medicine.

## 2 Methods

In conducting this scoping review, we followed the guidelines recommended by the Preferred Reporting Items for Systematic Reviews and Meta-Analyses Extension for Scoping Reviews (PRISMA-ScR) (17), as detailed in Supplementary Table 1.

### 2.1 Search strategies

Given the multidisciplinary nature of this scoping review, which spans both computer science and medical/healthcare fields, it was essential to select databases that comprehensively cover these areas. We systematically searched six databases: MEDLINE (via Ovid), EMBASE (via Ovid), CINAHL (via EBSCO), Scopus, IEEE Xplorer, and ACM Digital Library.

In the field of computer science, peer-reviewed conference papers are highly regarded, often on par with journal publications. As such, conference papers were included in this review, but abstracts without full-text papers were excluded.

The search terms were adapted for each database, and the full search strategy is detailed in Supplementary Table 2. The search strategy was structured around three conceptual blocks: (1) digital nudging and behaviour change techniques, (2) digital technologies, and (3) lifestyle-related health behaviours and conditions. Rather than using the single term “*lifestyle medicine*” as a search concept, we operationalised the lifestyle medicine domain through its constituent pillars, including nutrition, physical activity, sleep, mind-body health, avoidance of harmful substances, and social connection.

The term “*lifestyle medicine*” was intentionally not included as a mandatory search term because it is a relatively recent and inconsistently applied label across disciplines. Many interventions relevant to lifestyle medicine are indexed under specific behavioural or clinical topics (e.g., diet, exercise, sleep, smoking cessation) without being described as lifestyle medicine interventions. Requiring the term “*lifestyle medicine*” in the search could therefore have reduced sensitivity and excluded relevant studies. Instead, studies were subsequently assessed during screening against predefined eligibility criteria requiring alignment with one or more lifestyle medicine pillars.

Nevertheless, because some studies may be indexed primarily using the term “*lifestyle medicine*” without explicit reference to individual pillars, the possibility that a small number of relevant records were not retrieved cannot be excluded and is acknowledged as a limitation of this review.

### 2.2 Inclusion and exclusion criteria

Table 1 outlines the inclusion and exclusion criteria. The criteria were designed to ensure that only relevant research focusing on digital nudging techniques within the context of lifestyle medicine pillars, such as behaviour change, were included. Original research studies were included based on the presence of system implementation and evaluations. Conversely, studies that lacked focus on health-related behaviour change did not involve nudging techniques, or were non-peer-reviewed were excluded from the review. No restrictions were enforced regarding the date and country of publication.

**Table 1:** Inclusion and exclusion criteria.

Area	Inclusion criteria	Exclusion criteria
1. Lifestyle medicine	– Focus on lifestyle medicine pillars (e.g., nutrition/food, physical activities/exercises, mind-body health, sleep, avoidance of harmful substances/addiction, and connections/relationships), including behaviour change.	– Studies focusing solely on general behaviour change unrelated to health.
		– Disease-related studies without involving lifestyle medicine pillars in disease management.
		– Non-nudging-related behavior change studies.
2. Digital	– Describes system implementation and nudging techniques.	– Studies proposing system designs without implementation or evaluation.
3. Nudging	– Evaluate the nudging techniques, including their usability, accuracy, usage, and acceptability, and preferences) through qualitative, quantitative or a mixed evaluation method.	– Studies without explanation of technologies and their nudging techniques.
		– Reviews that did not explain nudging techniques or their efficacy.
4. Publication	– Original research and peer-reviewed studies, including conference proceedings.	– Non-English publications, review articles, conference abstracts, proposals, editorials, commentaries, and preprints.
	– Published in English.	– Letters to the editor, commentaries, or proposals without empirical evidence.

### 2.3 Study selection

We used Zotero version 6, an open-source tool, to collect, organise, annotate, screen, and select papers for this review. Search results from each database were downloaded as CSV files.

The study selection process involved three steps: (i) removing duplicates, (ii) screening titles and abstracts, and (iii) assessing eligibility. After removing duplicates, the remaining citations were screened by reviewing their titles and abstracts. After screening against the exclusion criteria, the full text of studies were then assessed.

Two reviewers independently performed the study selection process. Disagreements between them in the second and third steps were resolved through discussion. Cohen’s κ was calculated to measure the level of agreement between the two reviewers (18). The kappa values for the title and abstract screening are above 0.8, indicating perfect agreement between the reviewers.

### 2.4 Data extraction and synthesis

Data extraction was composed of two main parts: (i) the core details of each paper, which were downloaded from Zotero, and (ii) additional information gathered directly from the papers. Information such as the type of publication (journal article or conference paper), title, list of authors, abstract, keywords, venue, and year of publication. Relevant information on lifestyle medicine pillars addressed, the system developed or used in the study, the technology involved, the study domain, the nudging techniques employed, the number of participants in the trials, and the methods and targets for evaluation were captured. All of the extracted data was recorded in Google Sheets, and the data extraction components can be found in Supplementary Table 3.

The data gathered from the included studies were synthesised using a narrative approach. This involved summarising and describing the information through text, tables, and figures.

The studies were categorised into several key themes based on the nudging techniques used, such as one-way nudging, nudging through system and technology interactions, peer-based nudging, and self-nudging. We explored the systems and techniques in these studies, highlighting their strengths and limitations.

## 3 Results

### 3.1 Search results

**Figure 1:**
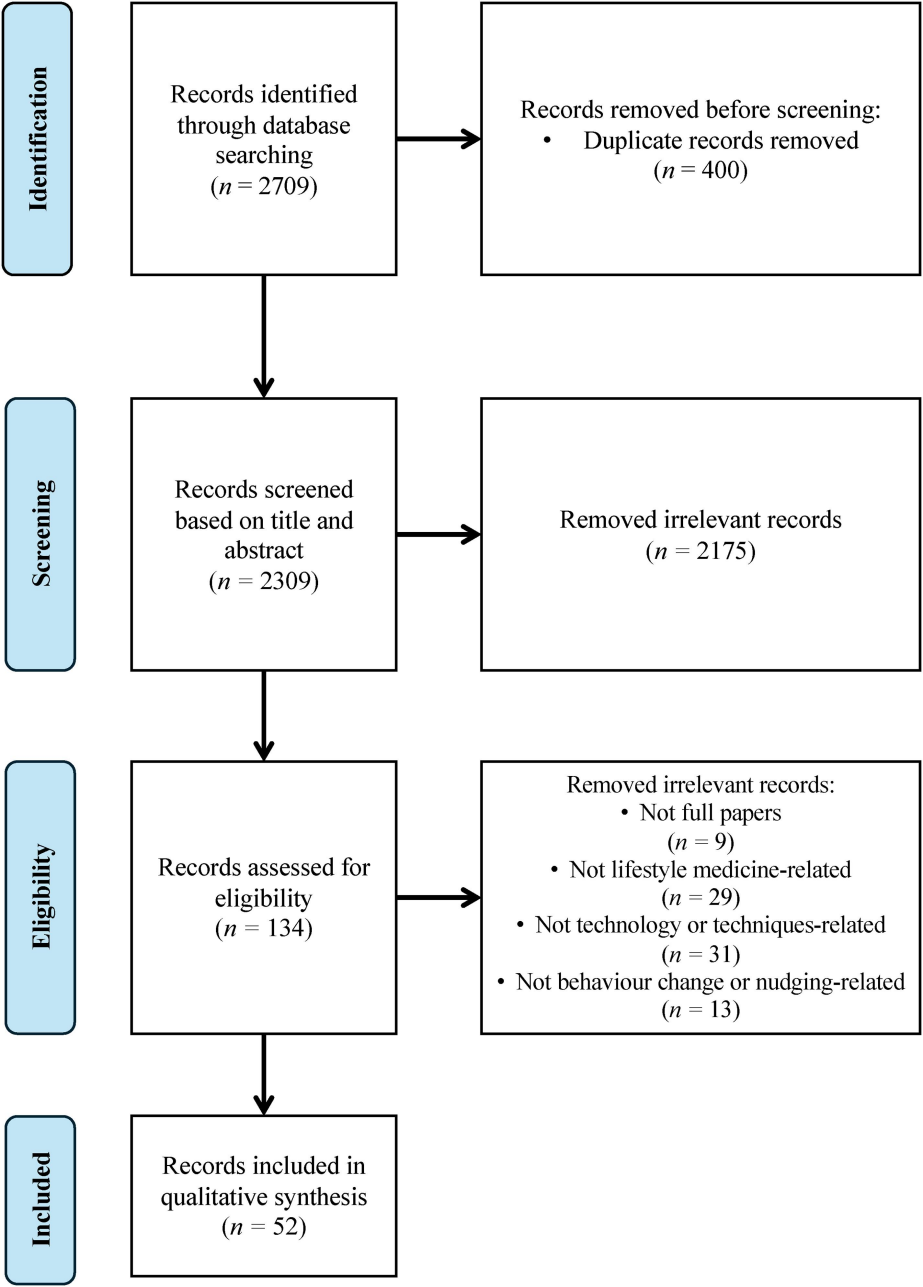
Flowchart of the study selection process using the PRISMA flow diagram.

An initial keyword search retrieved 2,709 articles. After removing 400 duplicates, 2,309 unique articles remained. Following a title and abstract screening, 2,175 articles were excluded. This left 134 articles for full-text assessment. After applying the eligibility criteria, 82 articles were excluded for reasons such as unavailability of the full paper (n=9), not being related to lifestyle medicine (n=29), not implementing digital technology or proposing nudging techniques (n=31), or lacking focus on behaviour change or nudging (n=13). Ultimately, 52 articles were included in the review. Figure 1 shows the PRISMA flow diagram for the study selection process.

### 3.2 Study characteristics

#### 3.2.1 Publication types, number of years, and domains

Digital nudging in lifestyle medicine bridges computer science and health or medical domains. A significant majority of the papers (n=40) synthesised in this scoping review originated from the computer science domain, with the remainder coming from the health and medical fields. In computer science, peer-reviewed conference papers are common, with top-ranked conferences being on par with high-impact journals. In line with this, most conference papers came from computer science (n=28). Given the rapid advancements in digital technology, most papers (n=37) were published in the last five years, and much less (n=3) were published more than a decade ago. Table 2 shows the publication types, years of publication, and their respective domains.

**Table 2:** Distribution of publications by type, publication year, and domain.

Publication type	Number of years	Computer Science	Health/Medical
Conference	<5 years	18	1
	5–10 years	9	2
	11–15 years	1	0
Journal	<5 years	9	9
	5–10 years	1	0
	11–15 years	2	0
Total		40	12

#### 3.2.2 Lifestyle medicine and technologies

Nutrition is the most frequently addressed lifestyle medicine pillar in digital nudging interventions. Half of the reviewed papers (n=26) focused on nutrition-related topics such as healthy eating, food choices, online grocery shopping, recipes, and snacks. Physical activity, including exercises, fitness routines, walking, and step counting, was the second most common area (n=14). Sleep was the least explored domain, with only one paper addressing it. Notably, no studies focused on the connection pillar of lifestyle medicine, such as connections with nature, animals, or society.

Three types of digital technologies have been utilised for nudging: (i) mobile apps and devices, (ii) web systems, and (iii) emerging technologies. Mobile apps and devices remain the most prevalent technologies for nudging purposes (n=22). Emerging technologies, such as virtual and augmented reality and various sensors (e.g., kinetic sensors, voice-activated data capture, smart speakers, voice-based user interfaces, and hand scanning devices), are gaining attention in the computer science field and are seeing increasing use in digital nudging (n=14).

Table 3 shows the distribution of digital technologies across lifestyle medicine domains.

**Table 3:** Distribution of number of studies of digital technologies across lifestyle medicine domains.

Lifestyle Medicine	Mobile app/device	Web system	Emerging technology
Nutrition	10	9	7
Physical activity	8	2	4
Mind-body health	0	2	1
Sleep	1	0	0
Addiction/harmful substance	3	3	2
Total	22	16	14

### 3.3 Thematic analysis of digital nudging

To better understand how digital nudging is implemented in lifestyle medicine interventions, we conducted an inductive thematic analysis of the included studies. Rather than applying a pre-existing digital nudging taxonomy, themes were derived from patterns observed across the included interventions. This analysis resulted in four categories of digital nudging: (i) one-way nudging, (ii) nudging through interactions with technology, (iii) nudging through peer interaction, and (iv) self-nudging.

**Figure 2:**
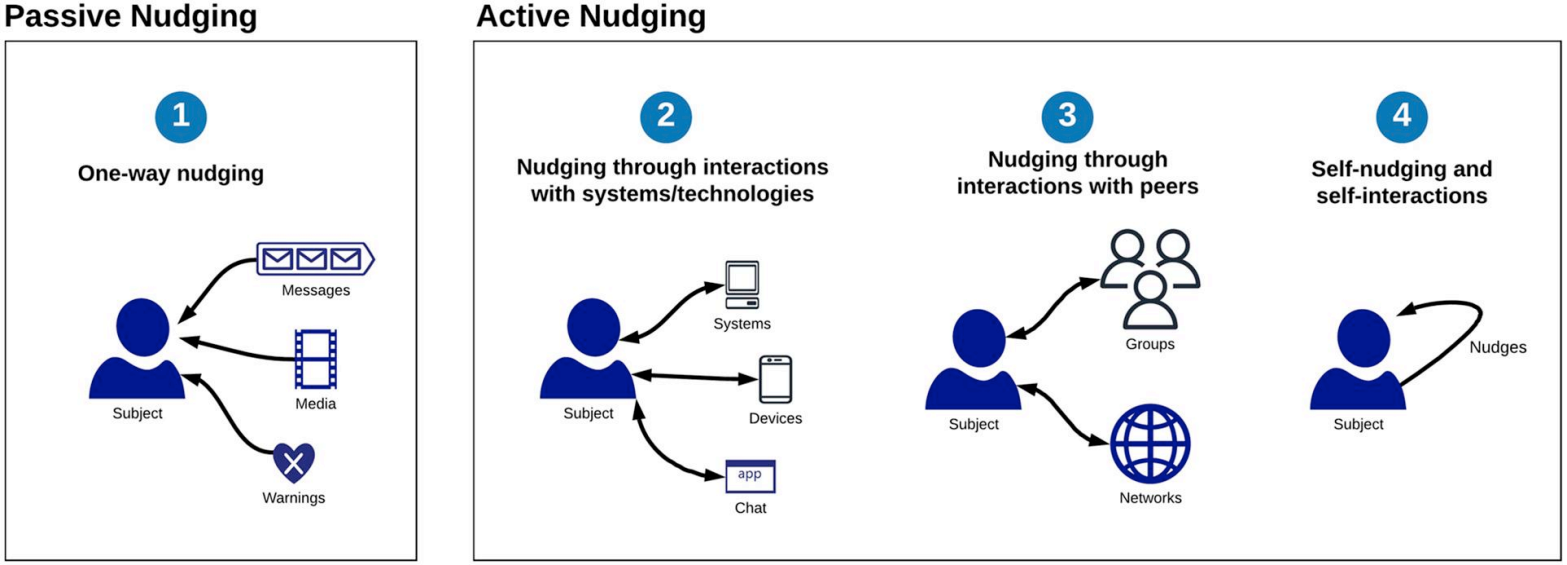
Passive and active nudging models.

From these emergent categories, we further developed a conceptual framework distinguishing between passive and active forms of digital nudging. Figure 2 illustrates this framework. In the passive nudging model, individuals primarily receive nudges without actively contributing to the nudging process (e.g., one-way nudging through reminders, notifications, or prompts). In the active nudging model, individuals actively participate in generating, reinforcing, or responding to nudges through interactions with technology, engagement with peers, or self-directed nudging activities. The passive/active distinction and the four digital nudging categories should therefore be interpreted as an inductively derived conceptual framework emerging from this review rather than as an established taxonomy.

The identified categories describe how nudging occurs rather than the specific technologies used to implement an intervention. Technologies such as mobile applications, web-based platforms, chatbots, augmented and virtual reality systems, wearable sensors, and artificial intelligence were not treated as separate digital nudging categories. Instead, they functioned as delivery platforms, interaction modalities, or enabling technologies that support one or more forms of digital nudging. Consequently, the same technology may facilitate multiple nudging categories within a single intervention.

Several specific nudging techniques were identified across the included studies. A *defaulting nudge* (or *default nudge*) refers to pre-selecting a preferred option while allowing users to opt out. A *labelling nudge* uses descriptive labels or indicators to guide decision-making. A *friction nudge* modifies the effort required to perform a behaviour, while a *swap nudge* encourages substitution of a less desirable option with a healthier or more desirable alternative. These techniques may be implemented across one or more of the digital nudging categories identified in this review.

Table 4 illustrates the relationship between these nudging types and various lifestyle medicine domains. The one-way nudging model is the most widely used, appearing in 23 studies, and applied across areas such as nutrition, physical activity, mind-body health, and addiction or harmful substances. Nutrition stands out as the most frequently addressed pillar of lifestyle medicine, featured in 26 studies, with interventions employing one-way nudging, technology-based nudging, and self-nudging. In contrast, mind-body health and sleep combined accounted for less than 10% of the studies (n=5). Moreover, the “connection” pillar of lifestyle medicine was not addressed in any of the digital nudging studies, and is therefore absent from the table.

**Table 4:** Number of studies showing the relationship between lifestyle medicine domains and types of digital nudging.

Lifestyle Medicine	One-way nudging	Nudging technologies	Nudging peers	Self-nudging
Nutrition	12	5	0	9
Physical activity	3	7	3	1
Mind-body health	1	2	1	0
Sleep	0	0	0	1
Addiction/Harmful substance	7	0	0	0
Total	23	14	4	11

### 3.4 One-way nudging

There are three types of one-way nudging: (i) Messages as nudges, (ii) Choice architecture as nudges, and (iii) Warnings as nudges.

#### 3.4.1 Messages as nudges

These are the simplest forms of nudging, typically delivered through text messages (19). In some cases, these messages were enhanced with visual aids (20) or video content (21) to increase their persuasiveness. The digital platforms used for these nudges ranged from mobile phones and apps to Microsoft Teams and social media platforms. While Kaptein et al. (19) focused on reducing snacking, Duro et al. (20), Teuber et al. (21), and Cherubini et al. (22) primarily targeted physical activity, particularly walking.

Messages as nudges produced mixed outcomes across different applications. Tailored Short Messaging Services (SMS) employing social influence approaches supported reduced unhealthy snacking behaviour. Motivational messages supplemented with infographics or incentives showed potential for enhancing motivation toward physical activity, though several studies reported limited evidence of maintained behaviour modification over time. Daily SMS reminders containing video links designed to promote physical activity breaks demonstrated minimal influence, with no substantial improvement detected among remote workers.

Section 1 of Table 5 summarises the strengths and limitations of messages as nudges.

**Table 5:** One-way nudging techniques.

Nudging technique	LM domain	Strength	Limitation	Ref
1. Messages as nudges				
Socially-tailored SMS	Nutrition	Reduced snacking	None reported	(19)
Motivational SMS with visuals	Physical activity	Increased motivation	No behaviour data	(20)
Motivational SMS with incentives	Physical activity	Boosted short-term motivation	Weak long-term effect	(22)
Daily SMS with physical activity videos	Physical activity	Tested on remote workers	No PA change	(21)
2. Choice architecture nudges				
Product ordering, defaulting, ranking, simplification	Food	Increased healthy food selection (e.g., high-fibre or low-sodium products)	Context-dependent and sometimes limited to short-term impacts.	(23–27)
Nutri-score, eco-score, and menu labelling	Food	Consistently improved nutritional quality of food choices across lab and real-world studies.	Eco-scores and labels may have weaker effects on their own compared to when combined.	(28–30)
Visual blocking (e.g., opaque layer) with display techniques	Food	Reduced visibility of unhealthy items and boosted social engagement via public displays.	Display nudges may require supplementary transparency nudges to maintain their intended outcomes.	(31, 32)
3. Warning as nudges				
Sensory feedback (vibrations, chirps, body detection, signage)	Addiction / Smoking / PA	Reduced digital overuse, sedentary behaviour, and public smoking incidents.	Risk of users ignoring the nudges over time (habituation).	(33–36)
Friction, pause-reminder, blocking, and default nudges	Addiction	Helped reduce excessive social media or screen usage and raised user awareness.	Success depends on user willingness to comply; some users found them annoying or disruptive	(37–39)
Warning labels with swap options	Nutrition / Addiction	Successfully nudged users toward healthier or safer purchases (e.g., alcohol reduction).	More impactful when combined with additional nudges (e.g., defaults or incentives).	(40, 41)

#### 3.4.2 Choice architecture nudging

This type of nudging primarily focuses on nutrition by reshaping the environment in which decisions are made. These strategies were applied through various platforms, including online grocery systems (23, 24, 26, 29, 31, 42), recipe recommendation tools (25, 28)), and lunch ordering systems (27, 30, 32). The majority of these studies used web-based platforms, except for Chang et al. (32), which used a large digital public display alongside a personal mobile app. Choice architecture nudging was also successfully implemented in primary and high school canteens, as well as in office lunch environments (27, 30, 32).

The use of choice architecture appears highly effective in guiding healthier food decisions, particularly within online grocery and school canteen settings. Techniques such as product ordering, defaulting, and simplified choices consistently encouraged healthier purchases across multiple studies. Similarly, Nutri-score and eco-score labelling proved valuable, especially when combined with prompts or social cues. However, some limitations emerged: effects were often context-dependent and sometimes diminished when nudges were used in isolation, such as eco-score labels alone. Display-focused nudges, like visual blocking of unhealthy items, showed promise but often required supplementary strategies (e.g., transparency messaging) to maximise impact.

Section 2 of Table 5 summarises the strengths and limitations of choice architecture as nudges.

#### 3.4.3 Warnings as nudges

*Warnings as nudges* were primarily aimed at addressing addiction, such as social media addiction and digital well-being (14, 33, 37–39), as well as harmful substance use, like smoking and alcohol consumption (34, 35, 40). Additionally, warnings were used in nutrition and physical activity interventions (36, 41). To reduce screen time, several techniques were employed, including phone vibrations, chirping, blocking or removing content, reminder and feedback nudges, and reward and punishment mechanisms (14, 33, 37–39). Warnings were also used to discourage harmful behaviours such as smoking and alcohol consumption (34, 40). Reminders and notifications were also used to promote healthy habits, particularly in nutrition and physical activity (36, 41). These reminders were delivered through various systems, offering warnings in different formats and frequencies.

Warning-based nudges demonstrated promising results across domains such as addiction, nutrition, and physical activity. Sensory feedback techniques (e.g., vibrations, signage) were effective but risked being ignored over time. Friction-based nudges and reminders helped reduce screen overuse but were sometimes seen as annoying, limiting user compliance. Labelling and swap nudges positively influenced healthier purchasing behaviours, though they worked best when paired with other supportive strategies.

Section 3 of Table 5 summarises the strengths and limitations of warnings as nudges. The details of each study on one-way nudges, covering messages as nudges, choice architecture as nudges, and warnings as nudges, can be found in Supplementary Table 4.

### 3.5 Nudging using interactions with technology

Nudging behaviours through interactions with technology often involve the use of two types of technologies: (i) Emerging technologies, and (ii) Chatbots.

#### 3.5.1 Emerging technologies

Emerging technologies such as augmented reality (AR), virtual reality (VR), voice recognition, and sensor-based devices have been widely applied in digital nudging interventions. For instance, AR was used to support home yoga workouts through head-mounted displays (43), while VR facilitated physical activity and virtual exploration in aged-care settings (44). Voice recognition tools, such as Amazon Alexa, enabled users to track home-based exercises like sit-ups and push-ups (45). In the nutrition domain, digital augmentation and sensor-embedded utensils were employed to promote healthier eating habits. Motion-tracking and projected lighting helped make food more appealing to children (46), while smart chopsticks with sensors translated eating patterns into digital art to encourage balanced diets (47, 48).

Spatial and location-based technologies were also leveraged to reduce unhealthy behaviours. For example, geofencing alerts and in-store hand-scanning devices were used to prompt healthier food choices in military and supermarket settings (49, 50). Additionally, Voice User Interfaces (VUI) supported smoking cessation by enabling users to log cravings and access mindfulness exercises via voice commands (51).

Overall, emerging technologies showed promising results in promoting physical activity, healthier eating behaviours, and harm reduction. AR and VR overlays enhanced exercise engagement but faced usability challenges due to equipment requirements. Voice-based interactions and smart utensils offered engaging, real-time feedback for behaviour monitoring, while spatial nudges provided timely environmental prompts. Despite these strengths, limitations such as novelty effects and long-term engagement concerns were observed.

Section 1 of Table 6 summarises the strengths and limitations of nudging using emerging technologies.

**Table 6:** Nudging techniques using emerging technologies.

Nudging technique	LM domain	Strength	Limitation	Ref
1. Nudging using Emerging Technologies				
Augmented Reality (AR)/Virtual Reality (VR) trainer-anchored overlays	Physical activity	Enhanced user engagement and exercise adherence.	Hardware discomfort (HMD weight) and limited accessibility.	(43, 44)
Voice-interactive self-monitoring systems	Physical activity/Smoking	Real-time monitoring and feedback (e.g., logging cravings or tracking exercises).	Dependent on voice recognition accuracy and user engagement.	(45, 51)
Sensor-enabled smart utensils with interactive feedback	Nutrition	Promoted mindful eating and slower consumption.	Limited sample sizes and novelty effect.	(46–48)
Geofenced mobile notifications with real-time nudges	Nutrition	Context-aware push notifications influenced healthier food choices.	Dependent on location-specific factors and user receptivity.	(49, 50)
2. Coaching and conversations with chatbot				
Conversational fitness coaching app with exercise plans and virtual training	Physical activity	Engagement and sustained use for physical activity.	Limited to short-term use.	(52)
AI-adaptive goal setting and progress monitoring via NutriWalking app	Physical activity	Supported regular aerobic exercise.	Requires ongoing engagement.	(53)
Human-expert-simulated conversational fitness coaching system	Physical activity	Enhanced motivation through expert feedback.	Human-in-the-loop limits scalability.	(54)
Story-based chatbot system using visual feedback and narrative-driven chapters	Physical activity	Encouraged self-reflection and sustained engagement.	Pilot study with small samples.	(55, 56)

#### 3.5.2 Chatbot-based nudging

Chatbots were used to promote physical activity (52–56). These systems offered structured guidance, facilitated social interactions, and provided virtual training sessions to enhance motivation and performance. For instance, Marcu et al. (52) developed a mobile app to encourage breast cancer survivors to increase their physical activity, while Mohan et al. (53) created an interactive coaching platform targeting overweight individuals with sedentary lifestyles to promote regular exercise. Vardhan et al. (54) designed an automated, personalised coaching system that engaged users through mini-conversations, and Murnane et al. (55, 56) used visual and textual narrative structures to inspire users, providing story-based visual feedback to track their activities and progress toward their goals.

Coaching and conversation-based chatbot interventions show potential in supporting physical activity, especially when integrated with personalised features such as tailored exercise plans, adaptive goal setting, and narrative feedback. Techniques using AI for adaptive coaching or expert-driven conversational agents enhance motivation. However, scalability challenges emerge when human experts participate, and long-term outcomes require further investigation in certain cases. Overall, chatbot-based nudging yields encouraging results for promoting physical activity, particularly through interactive and narrative components.

Section 2 of Table 6 summarises the strengths and limitations of using chatbots and conversational technologies for nudging. More detailed information on the technological nudging studies is available in Supplementary Table 5.

### 3.6 Nudging through interactions with peers

Nudging through peer interaction focused primarily on improving physical activity outcomes (57–60). Communication technologies, such as Google Docs and social messaging platforms, were leveraged to facilitate peer support, particularly for managing mental health concerns like depression and anxiety (57). In the physical activity domain, tracking technologies—including smartwatches, wristbands, and Fitbit devices—enabled peer-to-peer interactions by sharing fitness data such as step counts and activity levels (57–59). Additionally, social interaction and gamification elements, such as competition and cooperation, were employed to strengthen engagement and motivation.

Overall, peer-interaction-based nudging techniques showed promising results in encouraging behaviour change, particularly for increasing physical activity. Peer chats, guided communication, and collaborative features promoted group cohesion and social support, although some studies reported challenges such as peer pressure and fluctuating engagement levels. The visibility of peer fitness metrics and the integration of gamified elements successfully motivated users to increase activity, despite potential disengagement among less competitive individuals.

Table 7 summarises the strengths and limitations of nudging through peer interactions. The details of the extracted data from the studies are available in Supplementary Table 6.

**Table 7:** Peer-interaction-based nudging techniques.

Nudging technique	LM domain	Strength	Limitation	Ref
Peer chats and guided communication (group support, social interaction, messaging)	Physical activity	Fostered social support and group cohesion.	Risk of peer pressure and inconsistent participation.	(57)
Peer visibility of step counts and fitness metrics (incl. gamification, cooperation and competition)	Physical activity	Increased activity levels via social comparison.	May cause disengagement in less competitive users.	(58–60)

### 3.7 Self-nudging and self-interactions

Self-nudging can be classified into: (i) Self-tracking and self-observation as nudges, and (ii) Self-managing and self-monitoring as nudges.

#### 3.7.1 Self-tracking and self-observation as nudges

Self-tracking plays a vital role in promoting well-being by enabling individuals to monitor and reflect on their behaviours. Several studies have developed diverse self-logging systems for nutrition interventions. For example, Silva and Epstein (61) and Silva et al. (62) introduced a multimodal food journaling tool allowing users to log their dietary intake via database searches, text entries, photos, URLs, barcode scanning, and voice commands. Similarly, Cordeiro et al. (63) proposed a lightweight, photo-based food journaling approach, while Luo et al. (64) explored voice-based food logging. Valenčič et al. (65) focused on snack journaling with contextual details, such as time, location, and social setting. In addition, Bentley and Tollmar (66) and Bentley et al. (67) expanded self-logging to include physical activity, mood, and pain tracking.

Across these studies, self-nudging via self-tracking and self-observation demonstrated encouraging results. Multimodal logging approaches enhanced user flexibility and engagement, while photo-based and contextual journaling increased awareness of eating behaviours. Simplified logging techniques, combined with creative visual feedback (e.g., canvas paintings), supported reflection with minimal user effort. However, limitations such as reduced data detail and occasional incomplete logging were noted. Overall, self-tracking interventions were found to be effective or promising in supporting behaviour change.

Section 1 of Table 8 summarises the strengths and limitations of nudging using self-tracking and self-observation.

**Table 8:** Self-nudging techniques: self-tracking and self-observation.

Nudging technique	LM domain	Strength	Limitation	Ref
1. Self-tracking and self-observation				
Cross-platform food journaling supporting text, photo, voice, barcode, and database inputs	Nutrition	Enhanced logging flexibility and user preference matching.	Trade-offs between effort and logging completeness.	(61, 62)
Photo-based food logging with contextual metadata (location, time, social setting)	Nutrition	Increased awareness of snacking patterns and eating environments.	Limited logging to specific moments (snacks/meals).	(63, 65, 66)
Simplified dietary tracking using voice and quick-input methods, integrated with data aggregation and visual meal displays	Nutrition	Engaging visual feedback; low-effort logging.	Less granular data capture.	(64, 67)
2. Self-managing and self-monitoring				
AI-assisted reflective prompts with macronutrient analysis for food choice evaluation	Nutrition	Enhanced user awareness and reflection on food choices.	Dependent on user engagement and image accuracy.	(68, 69)
Activity tracker with goal-setting and feedback features for physical activity monitoring	Physical activity	Supported sustained self-monitoring behaviours.	User engagement declined over time.	(70)
Guided self-experimentation via feedback and personalisation	Sleep	Encouraged self-awareness and sleep habit adjustments.	High variability in individual user responses.	(71)

#### 3.7.2 Self-managing and self-monitoring as nudges

Self-nudging involves a proactive approach to *self-management, self-monitoring, and goal-setting*, where individuals actively shape their own behaviour by tracking and managing personal actions. Studies employed advanced technologies, such as machine learning and nutrient detection algorithms, to deliver personalised feedback and assist in setting goals across various lifestyle domains (68–71). These tools empowered users to self-assess, reflect on, and monitor their progress in areas such as nutrition, physical activity, and sleep.

Overall, self-managing and self-monitoring interventions showed promising results. Machine learning-driven reflection prompts and nutritional feedback heightened user awareness and supported behaviour change. Activity trackers with personalised and contextualised feedback contributed to sustained engagement in physical activity. Platforms guiding users through self-experiments also fostered greater autonomy, particularly in improving sleep habits. Despite these strengths, common limitations included challenges in maintaining long-term motivation and engagement.

Section 2 of Table 8 summarises the strengths and limitations of nudging using self-managing and self-monitoring techniques. The details of the extracted data from the studies are available in Supplementary Table 7.

## 4 Discussion

### 4.1 Nudges for lifestyle medicine

Most digital nudging studies included in this scoping review have centred around food choices and physical activity, both of which are essential pillars of lifestyle medicine. However, digital nudging has the potential to address a wider range of health-related behaviours. Broadening the research to include areas like sleep enhancement, stress management, and fostering social connections would enable a more comprehensive and integrated approach to promoting healthy lifestyle changes (72, 73).

Beyond the traditional pillars of lifestyle medicine, emerging wellness practices are also gaining traction. These include fostering connections with animals, nature, oneself, and with family and colleagues (74–76). Techniques such as deep breathing and diaphragmatic breathing, known for their effectiveness in managing stress and boosting mental clarity, are key examples (77, 78).

Future digital nudging initiatives must extend beyond individual behaviour change to address broader social determinants of health, such as access to social prescribing, housing, and employment support, ensuring that nudges promote not only health but also health equity (79, 80). This requires the development of culturally co-designed digital nudging platforms, created in collaboration with First Nations and culturally and linguistically diverse (CALD) communities, to ensure that techniques are contextually appropriate, respectful, and effective across diverse populations (81, 82).

### 4.2 Digital technologies used for nudging

Emerging technologies have primarily focused on influencing behaviour in areas such as nutrition and physical activity, with some promising applications in addiction prevention and mind-body health (46). However, the use of emerging technology across the broader pillars of lifestyle medicine, such as sleep, stress management, and social connection, remains underexplored. For digital nudging to be widely adopted as an effective tool for behaviour change, more research and development are necessary to extend emerging technology’s reach into these other pillars. Incorporating emerging technology into these overlooked areas of lifestyle medicine could open up new pathways for technology-assisted health interventions and significantly broaden the scope of digital nudging.

One of the key limitations of current emerging technology is its practicality. Many of these technologies are still in the experimental phase and are often bulky, complex, or impractical for daily use outside of controlled trials (43). For instance, using virtual reality (VR) for elderly patients or chopsticks embedded with sensors to track eating habits may have limited real-world applications (44, 47, 48). These tools are often difficult to integrate into everyday life, making them less feasible for widespread use. Moreover, implementing emerging technology is far more complex than traditional nudging techniques. Technologies such as Google Glass and robotic companions, like pet robots for emotional support, are examples of promising emerging technology, but they require extensive research, refinement, and testing to be truly effective in promoting behaviour change (83–86).

Even when advanced technologies are fully developed, there is no guarantee that people will continue to use them. For example, smoking cessation tools that track user behaviour may see high engagement initially, but after a month, many users may stop using the technology altogether (51). This highlights a key issue with emerging technology: adoption does not necessarily translate to sustained usage. More usability research is crucial to ensure that these technologies not only attract users but also encourage long-term engagement. Additionally, it is essential to determine whether these technologies are genuinely effective in altering health-related behaviours. Without understanding the usability and long-term effectiveness, many emerging technologies may fall short of their potential.

To maximise the impact of emerging technology, it is important to incorporate these technologies into existing health-related systems rather than treating them as stand-alone solutions. For instance, online grocery stores already use choice architecture to guide consumer behaviour. This existing framework could be further enhanced by integrating emerging technologies. For example, hand-scanning technology is already being utilised, but other sensors, like geofencing in physical supermarkets, could be introduced to nudge consumers toward healthier choices (49, 50). In online settings, AI recommendation systems could offer personalised nudges based on browsing habits and health data, creating a more seamless and integrated user experience to generate a healthier lifestyle, nutrition and movement plan.

Emerging technology is not limited to physical devices. AI technologies, such as Large Language Models (LLMs) and Generative AI (GenAI) tools like ChatGPT, are also ready to revolutionise health interventions (87–89). These AI systems could be leveraged for personalised health coaching, providing tailored advice, motivation, and feedback based on user data. By integrating AI into health and wellness platforms, nudging could become even more personalised, adaptive, and responsive to individual needs. However, research and trials are necessary to explore the efficacy of these technologies in real-world settings. Future investigations should focus on how AI-driven nudging could influence long-term behaviour change and support the broader goals of lifestyle medicine.

### 4.3 From system development to behaviour change

The development of digital nudging technologies follows a structured pipeline that progresses through distinct stages. Initially, the focus is on system development, where the accuracy and functionality of the technology are paramount. Once the system’s technological foundations are solidified, the next phase involves system trials that assess usability, feasibility, and how well the system aligns with the target behaviour. The final stage consists of larger trials, where the emphasis shifts toward measuring the impact of the nudges on actual behaviour change and health outcome metrics

Currently, about one-third of the studies on digital nudging covered in this scoping review remain centred on system usability, feasibility, accuracy, and acceptability rather than focusing solely on promoting behaviour change. While these are important steps in the development process, it is crucial that future research moves beyond system evaluation to prioritise health-related behaviour change outcomes. Ultimately, the success of digital nudging relies on whether it effectively drives the desired changes in behaviour, which should be the primary goal.

Moreover, over half the studies had <50 participants. This significantly limits generalizability and effect size reliability. To draw more meaningful conclusions and enhance the generalisability of results, there is a pressing need to expand trials into full randomised controlled trials (RCTs) with larger sample sizes (90). This would provide more robust evidence of the effectiveness of digital nudging in achieving its health-related behaviour change objectives.

### 4.4 Personalisation and role of AI

The studies reviewed often relied on participant interviews before the start of the trials to establish a baseline (19, 33, 36, 43, 51, 59). This self-reported baseline, gathered through interviews, serves as a point of comparison for the intervention phase. However, it is typically not comprehensive and fails to reflect the full profile of the participants. Additionally, in the intervention phase, the nudging strategies are not tailored to the individual profiles of the participants. This limited baseline is inadequate to serve as a true representation of the participants’ characteristics, making it ineffective for targeted behavioural interventions. To truly improve behavioural outcomes, nudging strategies must be more personalised, taking into account detailed user profiles.

To effectively personalise nudges, more comprehensive data collection methods are necessary to build an accurate profile of each user. There are various ways to gather data, both automatically and semi-automatically, using sensors, health-related data, and behavioural metrics (91). For instance, wearables can track physical activity, sleep patterns, and heart rate, while smartphone apps can monitor dietary habits, social interactions, and stress levels (92–95). These data points create a more holistic profile of the user, which can then be used to tailor the nudging interventions more effectively. By incorporating a broader range of personal information, such as physical and behavioural data, the nudging process becomes significantly more aligned with the needs and habits of the individual, making the interventions more impactful.

Personalisation has long been applied in health-related fields such as personalised nutrition and personalised medicine. In personalised nutrition, also known as precision nutrition (96–99), dietary recommendations are tailored based on an individual’s phenotype, which includes factors such as metabolic rate, genetics, gut microbiome, and other biological markers. By aligning nutritional advice with the individual’s unique biological makeup, these interventions can more effectively promote healthier eating habits and prevent diet-related chronic conditions like diabetes or cardiovascular disease.

Similarly, personalised (or precision) medicine adapts medical treatment plans based on a patient’s genetic profile and other factors such as lifestyle, medical history, and even environmental influences (100–102). For example, pharmacogenomics uses genetic information to predict how an individual will respond to certain medications, ensuring that treatments are both effective and free from harmful side effects. This level of customisation has transformed traditional healthcare into more precise, patient-centred care.

In non-health contexts like e-commerce and social media, personalisation has become the norm, with recommendation systems providing a highly individualised digital experience (103, 104). In online shopping, platforms use past purchases, browsing history, and user reviews to recommend products tailored to each customer’s preferences. Similarly, social media platforms like TikTok and Instagram utilise AI-driven algorithms to suggest content based on users’ past interactions, such as the videos they’ve watched, the accounts they follow, and the topics they engage with. These recommendation systems create a more personalised user experience by understanding and predicting user behaviour based on comprehensive data profiles.

In the context of digital nudging, similar AI algorithms can be employed to create personalised interventions, much like those used in e-commerce or social media recommendation systems. By integrating personal data, nudges can be customised to match the unique profiles and needs of each user. However, the effectiveness of these personalised nudges depends on the accuracy and comprehensiveness of the data collected. The more complete the user profile, incorporating not only basic demographic data but also behavioural, psychological, and health-related metrics, the more precise the nudging strategies can be. Therefore, a comprehensively designed data collection system is critical for enhancing the accuracy and efficacy of AI-driven personalised nudging.

### 4.5 Comparison with other scoping reviews or systematic reviews

Compared to existing systematic and scoping reviews, our study offers a broader and more integrative perspective by covering multiple lifestyle medicine (LM) pillars and focusing on both the techniques and the effectiveness of digital nudging interventions.

Previous reviews, such as those by Forberger et al. (15) and Benthem de Grave et al. (16), focused on specific LM domains. Forberger et al. (15) concentrated on workplace interventions aimed at promoting physical activity and reducing sedentary behaviour, predominantly through stair prompts and basic digital nudges. Similarly, Benthem de Grave et al. (16) examined smartphone apps for healthy and sustainable food purchasing, addressing challenges in app design and user engagement. While both reviews contribute valuable insights, they are limited to individual LM pillars (physical activity or nutrition) and did not explore the broader spectrum of LM domains as this review does.

In contrast, Valta et al. (12) and Sadeghian and Otarkhani (13) reviewed digital nudging more generally across various application contexts. Valta et al. (12) provided a taxonomy of digital nudging forms and contexts, while Sadeghian and Otarkhani (13) emphasised data-driven nudging and personalisation using AI and machine learning techniques. However, both reviews did not specifically address LM pillars or health behaviour change outcomes, focusing instead on digital nudging concepts and system designs.

Our scoping review fills this gap by systematically analysing nudging techniques across all major LM pillars (nutrition, physical activity, sleep, addiction, and mind-body health). Additionally, it evaluates the effectiveness of nudging approaches, providing actionable insights for LM interventions and digital health research.

### 4.6 Limitation

This scoping review has several limitations. First, our search strategy was restricted to English-language publications and did not extend to clinical trial registries (e.g., ClinicalTrials.gov). Consequently, we may have overlooked relevant studies, including non-English publications from technologically advanced regions and ongoing trials that employ basic technologies like SMS. Furthermore, a potential for publication bias must be acknowledged, particularly if industry-funded studies are more likely to report positive results, which could skew the overall body of evidence presented in this review.

Second, the synthesis of findings was challenged by significant heterogeneity in outcome measures across the included studies. Researchers assessed digital nudges using a wide spectrum of endpoints, from system usability and feasibility to behaviour change. This variability made direct comparison of outcomes exceedingly difficult. Related to this is the lack of a systematic framework to holistically assess digital health applications; studies often focused on a single aspect (e.g., engagement) without evaluating the entire pathway from nudge delivery to final health impact.

Finally, the assessment of individual study quality was constrained by the diversity of methodologies (e.g., RCTs, pilot studies, qualitative work) and the absence of a unified tool for evaluating such breadth. For instance, tools like the Mixed Methods Appraisal Tool (MMAT) (105), designed to appraise the methodological quality of diverse study types, were not employed in this review but would be valuable for future systematic analyses.

## 5 Conclusion

This scoping review has provided a comprehensive overview of digital nudging techniques within lifestyle medicine, addressing a gap in the literature that often focuses on narrower aspects or specific technologies. By bridging computer science and health domains, this review highlights the interdisciplinary nature of digital nudging and its applications in promoting health-related behaviour change.

The findings underscore the potential of digital nudging interventions, while also highlighting the need for larger sample sizes and more robust study designs, particularly randomised controlled trials, to strengthen the evidence base. Future research should focus on expanding trials to include more diverse populations and exploring the integration of AI and other advanced technologies to enhance personalisation and effectiveness.

Overall, this review demonstrates the significant promise of digital nudging techniques, especially those leveraging AI, in supporting lifestyle medicine and behaviour change. Continued interdisciplinary collaboration and rigorous research will be essential to fully realise the benefits of these innovative interventions.

## Data Availability

The original contributions presented in the study are included in the article/Supplementary Material. Further inquiries can be directed to the corresponding author.
