# Supplementary material for: Digital nudging techniques for behaviour change in lifestyle medicine: a scoping review
Source: Front Digit Health. 2026 Jul 30;8:1799205. doi: 10.3389/fdgth.2026.1799205 (PMC13470668; doi:10.3389/fdgth.2026.1799205)
Supplement: Supplementary file 1 [file Datasheet1.pdf]

Supplementary Table 1: Prisma-ScR Checklist

Table 1: Prisma-ScR Checklist

SECTION	ITEM	PRISMA-ScR CHECKLIST ITEM	REPORTED IN SECTION
**TITLE**			
Title	1	Identify the report as a scoping review	Title page
**ABSTRACT**			
Structured summary	2	Provide a structured summary that includes (as applicable): background, objectives, methods, results, and conclusions that relate to the review questions and objectives.	Abstract
**INTRODUCTION**			
Rationale	3	Describe the rationale for the review in the context of what is already known. Explain why the review questions/objectives lend themselves to a scoping review approach.	1. Introduction
Objectives	4	Provide an explicit statement of the questions and objectives being addressed with reference to their key elements (e.g., population or participants, concepts, and context) or other relevant key elements used to conceptualize the review questions and/or objectives.	1. Introduction
**METHODS**			
Protocol and registration	5	Indicate whether a review protocol exists; state if and where it can be accessed (e.g., a Web address); and if available, provide registration information, including the registration number.	N/A
Eligibility criteria	6	Specify characteristics of the sources of evidence used as eligibility criteria (e.g., years considered, language, and publication status), and provide a rationale.	2. Methods (section 2.2)

Table 1: Prisma-ScR Checklist (continued)

SECTION	ITEM	PRISMA-ScR CHECKLIST ITEM	REPORTED IN SECTION
Information sources	7	Describe all information sources in the search (e.g., databases with dates of coverage and contact with authors to identify additional sources), as well as the date the most recent search was executed.	2. Methods (section 2.1)
Search	8	Present the full electronic search strategy for at least 1 database, including any limits used, such that it could be repeated.	2. Methods (section 2.1)
Selection of sources of evidence	9	State the process for selecting sources of evidence (i.e., screening and eligibility) included in the scoping review.	2. Methods (section 2.3)
Data charting process	10	Describe the methods of charting data from the included sources of evidence (e.g., calibrated forms or forms that have been tested by the team before their use, and whether data charting was done independently or in duplicate) and any processes for obtaining and confirming data from investigators.	2. Methods (section 2.3)
Data items	11	List and define all variables for which data were sought and any assumptions and simplifications made.	2. Methods (section 2.4)
Critical appraisal of individual sources of evidence	12	If done, provide a rationale for conducting a critical appraisal of included sources of evidence; describe the methods used and how this information was used in any data synthesis (if appropriate).	N/A
Synthesis of results	13	Describe the methods of handling and summarizing the data that were charted.	2. Methods (section 2.4)
**RESULTS**			
Selection of sources of evidence	14	Give numbers of sources of evidence screened, assessed for eligibility, and included in the review, with reasons for exclusions at each stage, ideally using a flow diagram.	3. Results (section 3.1)

Table 1: Prisma-ScR Checklist (continued)

SECTION	ITEM	PRISMA-ScR CHECKLIST ITEM	REPORTED IN SECTION
Characteristics of sources of evidence	15	For each source of evidence, present characteristics for which data were charted and provide the citations.	3. Results (section 3.2)
Critical appraisal within sources of evidence	16	If done, present data on critical appraisal of included sources of evidence (see item 12).	N/A
Results of individual sources of evidence	17	For each included source of evidence, present the relevant data that were charted that relate to the review questions and objectives.	3. Results (section 3.2)
Synthesis of results	18	Summarize and/or present the charting results as they relate to the review questions and objectives.	3. Results (section 3.3)
**DISCUSSION**			
Summary of evidence	19	Summarize the main results (including an overview of concepts, themes, and types of evidence available), link to the review questions and objectives, and consider the relevance to key groups.	4. Discussion
Limitations	20	Discuss the limitations of the scoping review process	4. Discussion
Conclusions	21	Provide a general interpretation of the results with respect to the review questions and objectives, as well as potential implications and/or next steps.	5. Conclusions
**FUNDING**			
Funding	22	Describe sources of funding for the included sources of evidence, as well as sources of funding for the scoping review. Describe the role of the funders of the scoping review.	N/A

Supplementary Table 2: Search Strategy

Table 2: List of Queries used to search various databases

DATABASE	QUERY	RESULTS
MEDLINE via Ovid	(digital and nudg* and (behaviour* or behavior*)).mp. [mp=title, book title, abstract, original title, name of substance word, subject heading word, floating sub-heading word, keyword heading word, organism supplementary concept word, protocol supplementary concept word, rare disease supplementary concept word, unique identifier, synonyms, population supplementary concept word, anatomy supplementary concept word]	51 papers
EMBASE via Ovid	(digital and nudg* and (behaviour* or behavior*)).mp. [mp=title, abstract, heading word, drug trade name, original title, device manufacturer, drug manufacturer, device trade name, keyword heading word, floating subheading word, candidate term word]	82 papers
CINAHL Complete	Search Terms: (digital) AND (nudg*) AND (behaviour* OR behavior*) Search modes: Find all/any of my search terms Limiters: Publication Date: 19000101-20241231	21 papers
Scopus	Search within: Article title, Abstract, Keywords Search documents: (digital) AND (nudg*) AND (behaviour* OR behavior*)	349 papers
IEEE xplorer	(”All Metadata”:Digital) AND (”All Metadata”:Nudg*) AND (”All Metadata”:Behaviour* OR ”All Metadata”:Behavior*) Filter: Journals, Conferences, 1884-2024	16 papers
ACM Digital Library	[All: digital] AND [All: nudg*] AND [[All: behaviour*] OR [All: behavior*]]	2190 papers
**TOTAL**		2709 papers

Supplementary Table 3: Data extraction form

Table 3: Data Extraction Form

CONCEPT	DEFINITION
**Paper characteristics**	
Key	Unique ID assigned to each study
Publication type	Journal or conference paper or book chapter
Publication year	The year in which the study was published
Author	The complete list of authors of the paper
Title	Title of the paper
Publication title	The title of the journal or conference proceedings or book series
Automatic tags	Keywords of the paper
**Study characteristics**	
Lifestyle medicine pillars	Lifestyle medicine pillars addressed in the study
Disease	Disease addressed in the study
Digital nudging technique	Digital nudging techniques used in the study
Systems	Name of the system developed for or used in this study
Technology type	Mobile apps, desktop systems, etc
**Evaluation**	
Study design	Qualitative, quantitative or mixed method
Intervention type	Randomised controlled trials or non-randomised
Evaluation method	Methods to evaluate the study (e.g. interviews, statistical methods)
Evaluation metrics	Metrics used to evaluate the model's performance
Number of participants	Number of participants (breakdown of gender), min/max age of participants
Length of study	Length of study (in days, weeks or months)
System testing	Usability and accuracy results
Efficiency	Efficiency of the nudging techniques

Supplementary Table 4: One-way nudging

Table 4: One-way nudging

Study	Type of publication	Lifestyle medicine	Systems or technologies	Nudging techniques	Intervention	Evaluation metrics
**1. Text messages as nudges**						
Kaptein et al. (2012)	Journal	Diet or snacking	SMS on mobile phone	Four message styles (authority, consensus, commitment, and scarcity)	RCT involving 72 participants into 3 groups (tailored, contra-tailored, random) for 2-week program	Reduced snacking
Duro et al. (2019)	Conference	Exercise	Social media platforms and online forums	Motivational messages, mixed with visual presentation	160 participants on regular and non-regular exercise	Impact to exercise, reasons, platform source, context, and visual presentation
Teuber et al. (2022)	Journal	Physical activity	Microsoft Teams	Text messages with video instructions	57 university students, 10-day program during COVID pandemic study at home	Increased physical activity
Cherubini et al. (2020)	Journal	Physical activity	Motivational messages on smart phone	Giving incentives (e.g. fixed incentive, lottery incentive, motivational message, and control group)	RCT involving 208 participants (84M/124F) into 4 groups for 10 months	Number of walking steps

Table 4: One-way nudging (continued)

Study	Type of publication	Lifestyle medicine	Systems or technologies	Nudging techniques	Intervention	Evaluation metrics
**2. Choice architecture as nudges**						
Valencic et al. (2024)	Journal	Food	Online grocery store using Wix.com software	Changing food order to favour high fibre option	RCT involving 175 participants (46M/127F, 2 non-binary) into 2 groups (intervention and comparator)	Fibre and energy food contents, food price
Jesse et al. (2021)	Journal	Food	A mock-up online grocery system	Default selection, highlighting, warning, and social information	Two trials involving a total of 217 participants	Perceptions of difficulty and satisfaction, confidence in the selected recipe, effectiveness of nudges
Michels et al. (2023)	Journal	Food	A mock-up online grocery system	An opaque white layer was added to the representation of unhealthy food	RCT involving 600 participants into 6 groups	Proportion of healthy and unhealthy food products chosen, motives, appetite level, effectiveness of nudges
Starke et al. (2021)	Conference	Food and recipe	Online recipe system	Re-ranking based on sodium contents and personal preferences	17 people participated in 4 trials	User choice behaviour and satisfaction, the effect of personalisation, the impact of sodium ranking

Table 4: One-way nudging (continued)

**Study**	**Type of publication**	**Lifestyle medicine**	**Systems or technologies**	**Nudging techniques**	**Intervention**	**Evaluation metrics**
El Majjodi et al. (2022)	Conference	Food	An interactive recommender system to generate recipe recommendations	Nutritional information	600 people used the recipe recommendation system	Impact of nutrition label, user experience, effectiveness of digital nudges
De Bauw et al. (2022)	Journal	Food	A simulated e-grocery store	Display of nutri-score and eco-score	A single-blinder RCT involving 994 participants into 2 groups (treatment and no treatment groups) each with 4 different information levels (no information, product, basket, and social norm), participants selected food products for one meal	Nutritional quality index, environmental impact index, and behavioural shifts
Berger et al. (2020)	Conference	Food	A mock-up online grocery system	Default rules, simplification, and social norms	291 participants involved in a 5-month trial	Impact of the three digital nudging elements (default rules, simplification, and socio norms)

Table 4: One-way nudging (continued)

**Study**	**Type of publication**	**Lifestyle medicine**	**Systems or technologies**	**Nudging techniques**	**Intervention**	**Evaluation metrics**
Wyse et al. (2021)	Journal	Food	Existing web-based canteen ordering system	Default orders, menu labelling, incentive strategy, traffic light nutritional label	RCT with 17 schools involving 2200 students for a 12-month period	Primary nutritional content, purchasing behaviour, and canteen revenue
Delaney et al. (2024)	Journal	Food	A school canteen lunch ordering system	Menu labelling and prompts	An RCT involving 9 schools, 1331 high school students aged 12 and 18 years over three distinct 8-week periods and 15-month follow-up	Proportion of 'every day,' 'occasional,' and 'should not be sold' items purchased, nutritional content of lunch orders, revenue, menu composition
Chang et al. (2018)	Conference	Food	A mobile app and a large public display	Public large displays, food daily challenges, social comparison	A 3-week RCT involving 145 people	User participation, usability and user experience, nutritional awareness, social comparison, behavioural impact

Table 4: One-way nudging (continued)

Study	Type of publication	Lifestyle medicine	Systems or technologies	Nudging techniques	Intervention	Evaluation metrics
**3. Warnings as nudges**						
Okeke et al. (2018)	Conference	Screen time	Use of Facebook	Gentle phone vibration	3-week experiment with 50 participants in three groups (control n=16, rigid condition n=19, and personalised condition n=15)	Number of opens and length of each open
Purohit et al. (2020)	Conference	Social media addiction	A Google Chrome extension tool called *NUDGE*	Hiding nudge, default nudge intervention, creating friction, pause-reminder nudge, feedback nudge, unfollow nudge	Two weeks trial with 67 participants	Reduced time spent on Facebook
Zhang et al. (2022)	Conference	Screen time	A *Chirp* Twitter client for Android mobile phones	External supports (time limit dialogue and usage stats page) and Internal supports (reading progress indicator, feed filter, recommended tweet blocker, custom lists)	A four-week trial with 31 participants (15M/15F/1 non-binary) with an average age of 36 years	Twitter usage time

Table 4: One-way nudging (continued)

**Study**	**Type of publication**	**Lifestyle medicine**	**Systems or technologies**	**Nudging techniques**	**Intervention**	**Evaluation metrics**
Rooksby et al. (2016)	Conference	Screen time	*ScreenLife* app that records device use (iOS, Android, Mac, Windows)	A visual presentation showing usage data for each of the user's devices	A 28-day trial, 21 students (10M/11F), qualitative analysis	Device use time
Choi et al. (2024)	Conference	Digital food content addiction	*FoodCensor* (Android mobile app and Google Chrome extension)	Conceal filter buttons, video thumbnails, and mute food videos	RCT: 3 weeks, involving 22 participants with binge eating disorder (BED) or bulimia nervosa (BN)	System's detection accuracy, impact on exposure to food content (food video played, suggested videos, food content search, perceived impact)
Ohta et al. (2022)	Conference	Smoking	A digital signage reacting to smoking in the non-smoking zone	An interactive signage change when smoking is detected (still image to image, image to video, video to video)	15 participants to test the system	Accuracy of the system

Table 4: One-way nudging (continued)

**Study**	**Type of publication**	**Lifestyle medicine**	**Systems or technologies**	**Nudging techniques**	**Intervention**	**Evaluation metrics**
Vigh et al. (2024)	Conference	Alcohol	A prototype online grocery system	Warning signs: alcohol warning banner, no or low alcohol priority listing, unit sort function, unit warning label, and swap function	11 people completed the questionnaire	Users' perceptions of the warning signs
Kang & Wei (2021)	Conference	Meal and Exercise	*FITLIFE* (fictitious fitness app) reminders/notifications on iPhone	43 tracking reminders on 5 categories (e.g., breakfast, lunch, dinner, exercise/snack, general meal/water) and participant's own created reminders	Study 1 (n=135, age=18-64) and Study 2 (n=100, age=20-66) to test the tracking reminder system	Users' perception on the messaging type of each tracking reminder
Haile et al. (2020)	Conference	Sedentary behaviour, physical activity, and mental wellbeing	Welbot	Messages (stretching, screen breaks, exercise, mindfulness, hydration tips, breathing exercises, etc.), frequency (every 40 min or 12 nudges per workday)	A 3-week single-arm trial (baseline vs follow up), 41 participants (6M/35F)	Sitting time, physical activity, sedentary behaviour (whole day, working day), well-being (anxiety, depression)

Supplementary Table 5: Nudging through interactions with systems and technologies

Table 5: Nudging through interactions with systems and technologies

Study	Type of publication	Lifestyle medicine	Systems or technologies	Nudging techniques	Intervention	Evaluation metrics
**1. Nudging using emerging technologies**						
Jo et al. (2023)	Conference	Yoga	FlowAR: an augmented reality training system for yoga	Augmented reality technology (trainer-anchored and user-anchored)	Study 1 for baseline performance with 16 participants (8M/8F), and study 2 in a home-like setting with 12 participants (6M/6F)	Usability of the system comparing the user-anchored and trainer-anchored layouts
Bradwell et al. (2024)	Conference	Physical activity and relaxation	*MOTUS Adventure*, and *MOTUS Relieve*	Virtual reality for exercise and relaxation	7 elderly (1M/6F) lived in an aged-care setting	Perceived benefits, concerns of use and implementation, negative effects, user experience, usability, suitability, acceptability and adoption
Luo et al. (2020)	Conference	Exercise	*TandemTrack* app with Alexa voice-activated data capture	Voice data capture, multimodal feedback, and daily reminders	22 participants, 4 week trial (11M/11F)	System usage and exercise behaviour

Table 5: Nudging through interactions with systems and technologies (continued)

**Study**	**Type of publication**	**Lifestyle medicine**	**Systems or technologies**	**Nudging techniques**	**Intervention**	**Evaluation metrics**
Ganesh et al. (2014)	Conference	Eating	*FoodWorks*, a digital augmentation tool of food using webcams and projectors	Changing the colour of food, and playing food games	7 families with their children aged 3-9 years (n=8)	A qualitative approach evaluating how the children perceived foods on the plate
Nakaoka et al. (2021); Nakamura et al. (2023)	Conference, Journal	Eating	The *eat2pic* system: a sensor-equipped chopstick and two digital canvases	Mapping eating speed and colours of food into painting in two canvases (one-meal and one-week canvases)	Study 1: 20 participants (19M/1F), and study 2: 30 participants (22M/8F) over one-week duration to test the device	One-meal eat2pic: slow down while eating and enjoyment, One-week eat2pic: variety of food by colour
McGuirt et al. (2022)	Journal	Food	*EatWellNow* mobile app, Bluetooth beacons, geofencing technology, and a mock-up online supermarker	Geofencing push notification and messaging	2621 participants in a 1-month trial to test the geofencing system	System usage, geofencing analytics

Table 5: Nudging through interactions with systems and technologies (continued)

Study	Type of publication	Lifestyle medicine	Systems or technologies	Nudging techniques	Intervention	Evaluation metrics
van der Laan & Orcholska (2022)	Journal	Food purchase	Hand scanning device	Visibility nudge and descriptive nutritional labelling nudge	Hand scanning function of an existing smartphone supermarket application used across 45 stores in the Netherlands	Comparison of healthier and unhealthy product purchases
Kjaerulff et al. (2024)	Conference	Smoking cessation, mindfulness exercises	Voice-based VUI (Voice User Interface) using Google Assistant and augmented VUI, a blend of VIU and GUI (Graphical User Interface)	Log craving in real-time and offer seven mindfulness exercises	In a month-trial, it started with 23 smokers, dropping out to only 9 participants	System usage, smoking urges, and mindfulness

Table 5: Nudging through interactions with systems and technologies (continued)

Study	Type of publication	Lifestyle medicine	Systems or technologies	Nudging techniques	Intervention	Evaluation metrics
**2. Coaching and conversations with chatbot**						
Marcu et al. (2018)	Conference	Physical activity for breast cancer survivors	Bounce mobile app	Six features: balance exercise plan, cautionary warnings, multiple representations of data, social interaction, virtual exercise trainer, incremental levels and progress indicators	4 breast cancer survivors, average age = 35 years, average time since breast cancer treatment = 3 years), 3 week trial	Usage of the app (adoption and use)
Mohan et al. (2020)	Journal	Aerobic exercise and sedentary behaviour	*NutriWalking* iOS mobile app	AI-based goal setting (e.g. difficult yet attainable, specific, proximal), goal reporting using daily and weekly scheduling, goal revision using AI human-agent interactions	21 patients with chronic health condition (e.g. T2DM) evaluated the usability of the system in a 6-week observational study	Adaptability, suitability, usability, and behaviour change

Table 5: Nudging through interactions with systems and technologies (continued)

Study	Type of publication	Lifestyle medicine	Systems or technologies	Nudging techniques	Intervention	Evaluation metrics
Vardhan et al. (2022)	Conference	Walk	PACE (**P**ersonalised and **A**utomated physical activity **C**oaching **E**ngine)	A wizard-based approach by human fitness expert	A 21-day trial with 33 participants comparing PACE with human coaches	Coach-participant conversation, step counts
Murnane et al. (2020, 2023)	Conference, Journal	Physical activity	WhoIsZuki	13 chapters of story-based visual feedback	39 participants, aged between 21 and 69, 4-5 months committed to the trial	Physical activity levels, goal setting and completion

Supplementary Table 6: Nudging through peer interactions

Table 6: Nudging through interactions with peers

Study	Type of publication	Lifestyle medicine	Systems or technologies	Nudging techniques	Intervention	Evaluation metrics
O'Leary et al. (2018)	Conference	Mental Health	GoogleDocs	Peer support chats using guided and unguided chats	A 2-week experiments with 40 people experiencing mental illnesses, four chats per week for 2 weeks	Effect of chat tools on depression and anxiety
Esakia et al. (2018)	Conference	Fitness	The *FitAware* system with a Pebble smartwatch interface	Peer's physical activity records to encourage own step counts	27 participants distributed into 9 groups, 8-week physical activity promotion intervention	Physical activity levels
Ren et al. (2018)	Journal	Fitness	*Mi-band* wristband	Peer's physical activity records to encourage own step counts	10 pairs of participants, 3-week data collection	Daily steps, social bonding
Chen & Pu (2014)	Conference	Fitness	*HealthyTogether* mobile app and *FitBit* tracker	Social interaction and gamification (competition and cooperation)	18 pairs of participants, 2 weeks of trial	Physical activity and social connection

Supplementary Table 7: Self-Nudging and Self-Interactions

Table 7: Self-Nudging and Self-Interactions

Study	Type of publication	Lifestyle medicine	Systems or technologies	Nudging techniques	Intervention	Evaluation metrics
**1. Self-tracking and self-observation**						
Silva & Epstein (2021); Silva et al. (2023)	Conference, Journal	Food journaling for weight loss and eating mindfulness	*ModEat* (a multiplatform system for Android and iOS phones, computers, Amazon Alexa and Google Voice Assistant)	Food logging using simulated database search, text description input, photo input, URL input, scanning barcode, barcode input, and voice input	A two-week trial, 15 participants (6M/9F), aged 25-50 years	Efficiency of the system using a thematic analysis (granularity and specificity, amount, context and automatic food interpretation)
Cordeiro et al. (2015)	Conference	Food journaling	*DECAF: Diary of Emotion, Contexts, and Food)*: mobile app and website	A light-weight photo-based food journal	27 participants aged between 21 and 64 years (31M/13F) to use *DECAF* for between 4 and 8 weeks	Usage of the app, impact of light-weight journaling, value of photo, and differences by journaler food goal

Table 7: Self-Nudging and Self-Interactions (continued)

Study	Type of publication	Lifestyle medicine	Systems or technologies	Nudging techniques	Intervention	Evaluation metrics
Valenčič et al. (2023)	Journal	Food journaling, focusing on snack	*SnackTrack* mobile app	Photo journaling of snack, including additional information such as when, how, what occasion, and where	8-week trial, 188 participants	Relationship between background image and healthfulness of the snack
Bentley & Tollmar (2013); Bentley et al. (2013)	Conference, Journal	Food journaling	*Health Mashup* mobile app	Food and exercise self-logging at the end of each day	Pilot study (10 participants) and main study (60 participants), both one-month duration	Logging frequency
Luo et al. (2021)	Conference	Food journaling	*FoodScrap* with *OmniTrack* [1]	Speed input for meal and snack logging	11 participants (2M/9F), one week trial	Benefits of speech-based food journaling

[1] https://omnitrack.github.io/research

Table 7: Self-Nudging and Self-Interactions (continued)

Study	Type of publication	Lifestyle medicine	Systems or technologies	Nudging techniques	Intervention	Evaluation metrics
**2. Self-managing and self-monitoring**						
G. Mitchell et al. (2021)	Conference	Nutrition	GlucoGoalie	Self-tracking, self-managing, self-assess, self-review	8 participants with T2DM completed a 4-week study using GlucoGoalie	Usage of the system, self-discovery, goal feedback, understanding goals
Joachim et al. (2022)	Journal	Nutrition	Diabetes self-management platform (patient mobile app and clinician web app)	AI-based macronutrient detection	Two use cases (nutrition recommendation, and macronutrient detection algorithm)	Usage of the system
Gouveia et al. (2015)	Conference	Physical activity	Habito app released on Google Play Store	Goal setting, contextualising physical activity, textual feedback that keeps updating	A 10-month study with 256 users	Usage and usefulness of Habito
Daskalova et al. (2020)	Conference	Sleep	SleepBandits app released on Google Play Store	Guide flexible self-experiments	365 active users of SleepBandits for 3 months	Usage and usefulness of SleepBandits

References

Bentley, F., & Tollmar, K. (2013). The power of mobile notifications to increase well-being logging behavior. In *Proceedings of the SIGCHI Conference on Human Factors in Computing Systems*. New York, NY, USA: Association for Computing Machinery. doi:10.1145/2470654.2466140.

Bentley, F., Tollmar, K., Stephenson, P., Levy, L., Jones, B., Robertson, S., Price, E., Catrambone, R., & Wilson, J. (2013). Health Mashups: Presenting Statistical Patterns between Wellbeing Data and Context in Natural Language to Promote Behavior Change. *ACM Trans. Comput.-Hum. Interact.*, *20*. doi:10.1145/2503823. Publisher: Association for Computing Machinery Place: New York, NY, USA.

Berger, M., Nüske, N., & Müller, C. (2020). Digital nudging in online grocery stores - Towards ecologically sustainable nutrition. In *International Conference on Information Systems, ICIS 2020 - Making Digital Inclusive: Blending the Local and the Global*. Association for Information Systems.

Bradwell, H. L., Cooper, L., Baxter, R., Tomaz, S., Edwards, K. J., Whittaker, A. C., & Jones, R. B. (2024). Implementation of Virtual Reality Motivated Physical Activity via Omnidirectional Treadmill in a Supported Living Facility for Older Adults: A Mixed-Methods Evaluation.: Virtual reality to motivate physical activity for older adults. In *Proceedings of the CHI Conference on Human Factors in Computing Systems*. New York, NY, USA: Association for Computing Machinery. doi:10.1145/3613904.3642281.

Chang, K. S.-P., Danis, C. M., & Farrell, R. G. (2018). Lunch line: using public displays and mobile devices to encourage healthy eating in an organization. In *Proceedings of the 2014 ACM International Joint Conference on Pervasive and Ubiquitous Computing*. New York, NY, USA: Association for Computing Machinery. doi:10.1145/632048.2636086.

Chen, Y., & Pu, P. (2014). HealthyTogether: exploring social incentives for mobile fitness applications. In *Proceedings of the Second International Symposium of Chinese CHI*. New York, NY, USA: Association for Computing Machinery. doi:10.1145/2592235.2592240.

Cherubini, M., Villalobos-Zuñiga, G., Boldi, M.-O., & Bonazzi, R. (2020). The Unexpected Downside of Paying or Sending Messages to People to Make Them Walk: Comparing Tangible Rewards and Motivational Messages to Improve Physical Activity. *ACM Trans. Comput.-Hum. Interact.*, *27*. doi:10.1145/3365665. Publisher: Association for Computing Machinery Place: New York, NY, USA.

Choi, R., Park, S., Han, S., & Lee, S.-J. (2024). FoodCensor: Promoting Mindful Digital Food Content Consumption for People with Eating Disorders. In *Proceedings of the CHI*

Conference on Human Factors in Computing Systems. New York, NY, USA: Association for Computing Machinery. doi:10.1145/3613904.3641984.

Cordeiro, F., Bales, E., Cherry, E., & Fogarty, J. (2015). Rethinking the Mobile Food Journal: Exploring Opportunities for Lightweight Photo-Based Capture. In *Proceedings of the 33rd Annual ACM Conference on Human Factors in Computing Systems*. New York, NY, USA: Association for Computing Machinery. doi:10.1145/2702123.2702154.

Daskalova, N., Yoon, J., Wang, Y., Araujo, C., Beltran, G., Nugent, N., McGeary, J., Williams, J. J., & Huang, J. (2020). SleepBandits: Guided Flexible Self-Experiments for Sleep. In *Proceedings of the 2020 CHI Conference on Human Factors in Computing Systems*. New York, NY, USA: Association for Computing Machinery. doi:10.1145/3313831.3376584.

De Bauw, M., De La Revilla, L. S., Poppe, V., Matthys, C., & Vranken, L. (2022). Digital nudges to stimulate healthy and pro-environmental food choices in E-groceries. *Appetite*, *172*. doi:10.1016/j.appet.2022.105971.

Delaney, T., Jackson, J., Lecathelinais, C., Clinton-McHarg, T., Lamont, H., Yoong, S. L., Wolfenden, L., Sutherland, R., & Wyse, R. (2024). Long-Term Effectiveness of a Multi-Strategy Choice Architecture Intervention in Increasing Healthy Food Choices of High-School Students From Online Canteens (Click & Crunch High Schools): Cluster Randomized Controlled Trial. *Journal of Medical Internet Research*, *26*. doi:https://dx.doi.org/10.2196/51108.

Duro, L., Campos, P. F., Romão, T., & Karapanos, E. (2019). How do motivational text messages impact motivation to exercise? implications for the design of activity trackers. In *Proceedings of the 13th Biannual Conference of the Italian SIGCHI Chapter: Designing the next Interaction*. New York, NY, USA: Association for Computing Machinery. doi:10.1145/3351995.3352043.

El Majjodi, A., Starke, A. D., & Trattner, C. (2022). Nudging Towards Health? Examining the Merits of Nutrition Labels and Personalization in a Recipe Recommender System. In *Proceedings of the 30th ACM Conference on User Modeling, Adaptation and Personalization*. New York, NY, USA: Association for Computing Machinery. doi:10.1145/3503252.3531312.

Esakia, A., McCrickard, D. S., Harden, S., & Horning, M. (2018). FitAware: Mediating Group Fitness Strategies with Smartwatch Glanceable Feedback. In *Proceedings of the 12th EAI International Conference on Pervasive Computing Technologies for Healthcare*. New York, NY, USA: Association for Computing Machinery. doi:10.1145/3240925.3240926.

G. Mitchell, E., M. Heitkemper, E., Burgermaster, M., E. Levine, M., Miao, Y., L. Hwang, M., M. Desai, P., Cassells, A., N. Tobin, J., G. Tabak, E., J. Albers, D., M. Smaldone, A., & Mamykina, L. (2021). From Reflection to Action: Combining Machine Learning with Expert Knowledge for Nutrition Goal Recommendations. In *Proceedings of the 2021 CHI Conference on Human Factors in Computing Systems*. New York, NY, USA: Association for Computing Machinery. doi:10.1145/3411764.3445555.

Ganesh, S., Marshall, P., Rogers, Y., & O'Hara, K. (2014). FoodWorks: tackling fussy eating by digitally augmenting children's meals. In *Proceedings of the 8th Nordic Conference on Human-Computer Interaction: Fun, Fast, Foundational*. New York, NY, USA: Association for Computing Machinery. doi:10.1145/2639189.2639225.

Gouveia, R., Karapanos, E., & Hassenzahl, M. (2015). How do we engage with activity trackers? a longitudinal study of Habito. In *Proceedings of the 2015 ACM International Joint Conference on Pervasive and Ubiquitous Computing*. New York, NY, USA: Association for Computing Machinery. doi:10.1145/2750858.2804290.

Haile, C., Kirk, A., Cogan, N., Janssen, X., A.-M., G., & Macdonald, B. (2020). Pilot testing of a nudge-based digital intervention (Welbot) to improve sedentary behaviour and wellbeing in the workplace. *International Journal of Environmental Research and Public Health*, *17*, 1–13. doi:https://dx.doi.org/10.3390/ijerph17165763. Publisher: MDPI AG (Postfach, Basel CH-4005, Switzerland. E-mail: rasetti@mdpi.com) Place: Switzerland.

Jesse, M., Jannach, D., & Gula, B. (2021). Digital Nudging for Online Food Choices. *Frontiers in Psychology*, *12*. doi:10.3389/fpsyg.2021.729589.

Jo, H.-Y., Seidel, L., Pahud, M., Sinclair, M., & Bianchi, A. (2023). FlowAR: How Different Augmented Reality Visualizations of Online Fitness Videos Support Flow for At-Home Yoga Exercises. In *Proceedings of the 2023 CHI Conference on Human Factors in Computing Systems*. New York, NY, USA: Association for Computing Machinery. doi:10.1145/3544548.3580897.

Joachim, S., Forkan, A. R. M., Jayaraman, P. P., Morshed, A., & Wickramasinghe, N. (2022). A Nudge-Inspired AI-Driven Health Platform for Self-Management of Diabetes. *Sensors*, *22*. doi:10.3390/s22124620.

Kang, J., & Wei, L. (2021). Enter Your Dinner Now! Uncovering Persuasive Message Attributes in Tracking Reminders that Motivate Logging. In *Proceedings of the 14th EAI International Conference on Pervasive Computing Technologies for Healthcare*. New York, NY, USA: Association for Computing Machinery. doi:10.1145/3421937.3422014.

Kaptein, M., De Ruyter, B., Markopoulos, P., & Aarts, E. (2012). Adaptive Persuasive Systems: A Study of Tailored Persuasive Text Messages to Reduce Snacking. *ACM Trans.*

Interact. Intell. Syst., *2*. doi:10.1145/2209310.2209313. Publisher: Association for Computing Machinery Place: New York, NY, USA.

Kjaerulff, S. B., Pedersen, S. B., Sigvardsen, T. J., Van Berkel, N., & Papachristos, E. (2024). Exploring VUI-Supported Mindfulness Techniques for Smoking Cessation. In *Proceedings of the 6th ACM Conference on Conversational User Interfaces*. New York, NY, USA: Association for Computing Machinery. doi:10.1145/3640794.3665546.

van der Laan, L. N., & Orcholska, O. (2022). Effects of digital Just-In-Time nudges on healthy food choice – A field experiment. *Food Quality and Preference*, *98*. doi:10.1016/j.foodqual.2022.104535.

Luo, Y., Kim, Y.-H., Lee, B., Hassan, N., & Choe, E. K. (2021). FoodScrap: Promoting Rich Data Capture and Reflective Food Journaling Through Speech Input. In *Proceedings of the 2021 ACM Designing Interactive Systems Conference*. New York, NY, USA: Association for Computing Machinery. doi:10.1145/3461778.3462074.

Luo, Y., Lee, B., & Choe, E. K. (2020). TandemTrack: Shaping Consistent Exercise Experience by Complementing a Mobile App with a Smart Speaker. In *Proceedings of the 2020 CHI Conference on Human Factors in Computing Systems*. New York, NY, USA: Association for Computing Machinery. doi:10.1145/3313831.3376616.

Marcu, G., Misra, A., Caro, K., Plank, M., Leader, A., & Barsevick, A. (2018). Bounce: Designing a Physical Activity Intervention for Breast Cancer Survivors. In *Proceedings of the 12th EAI International Conference on Pervasive Computing Technologies for Healthcare*. New York, NY, USA: Association for Computing Machinery. doi:10.1145/3240925.3240927.

McGuirt, J. T., Gustafson, A., Ammerman, A. S., Tucker-Mclaughlin, M., Enahora, B., Moore, C., Dunnagan, D., Prentice-Dunn, H., & Bedno, S. (2022). EatWellNow: Formative Development of a Place-Based Behavioral "Nudge" Technology Intervention to Promote Healthier Food Purchases among Army Soldiers. *Nutrients*, *14*. doi:10.3390/nu14071458.

Michels, L., Ochmann, J., Schmitt, K., Laumer, S., & Tiefenbeck, V. (2023). Salience, transparency, and self-nudging: a digital nudge to promote healthier food product choices. *European Journal of Information Systems*, . doi:10.1080/0960085X.2023.2229787.

Mohan, S., Venkatakrishnan, A., & Hartzler, A. L. (2020). Designing an AI Health Coach and Studying Its Utility in Promoting Regular Aerobic Exercise. *ACM Trans. Interact. Intell. Syst.*, *10*. doi:10.1145/3366501. Publisher: Association for Computing Machinery Place: New York, NY, USA.

Murnane, E. L., Glazko, Y. S., Costa, J., Yao, R., Zhao, G., Moya, P. M. L., & Landay, J. A. (2023). Narrative-Based Visual Feedback to Encourage Sustained Physical Activity: A Field Trial of the WhoIsZuki Mobile Health Platform. *Proc. ACM Interact. Mob. Wearable Ubiquitous Technol.*, *7*. doi:10.1145/3580786. Publisher: Association for Computing Machinery Place: New York, NY, USA.

Murnane, E. L., Jiang, X., Kong, A., Park, M., Shi, W., Soohoo, C., Vink, L., Xia, I., Yu, X., Yang-Sammataro, J., Young, G., Zhi, J., Moya, P., & Landay, J. A. (2020). Designing Ambient Narrative-Based Interfaces to Reflect and Motivate Physical Activity. In *Proceedings of the 2020 CHI Conference on Human Factors in Computing Systems*. New York, NY, USA: Association for Computing Machinery. doi:10.1145/3313831.3376478.

Nakamura, Y., Nakaoka, R., Matsuda, Y., & Yasumoto, K. (2023). eat2pic: An Eating-Painting Interactive System to Nudge Users into Making Healthier Diet Choices. *Proceedings of the ACM on Interactive, Mobile, Wearable and Ubiquitous Technologies*, *7*. doi:10.1145/3580784. Publisher: Association for Computing Machinery.

Nakaoka, R., Nakamura, Y., Matsuda, Y., Misaki, S., & Yasumoto, K. (2021). Eat2pic: Food-tech Design as a Healthy Nudge with Smart Chopsticks and Canvas. In *2021 IEEE International Conference on Pervasive Computing and Communications Workshops and other Affiliated Events, PerCom Workshops 2021*. Institute of Electrical and Electronics Engineers Inc. doi:10.1109/PerComWorkshops51409.2021.9431002.

Ohta, T., Fujisawa, K., Fuminori, K., Kawaguchi, S., & Muramoto, R. (2022). Digital Signage that Reacts to Smoking in Non-Smoking Zone. *International Journal of Asia Digital Art and Design*, *26*. doi:10.20668/adada.26.4_37.

Okeke, F., Sobolev, M., Dell, N., & Estrin, D. (2018). Good vibrations: can a digital nudge reduce digital overload? In *Proceedings of the 20th International Conference on Human-Computer Interaction with Mobile Devices and Services*. New York, NY, USA: Association for Computing Machinery. doi:10.1145/3229434.3229463.

O'Leary, K., Schueller, S. M., Wobbrock, J. O., & Pratt, W. (2018). "Suddenly, we got to become therapists for each other": Designing Peer Support Chats for Mental Health. In *Proceedings of the 2018 CHI Conference on Human Factors in Computing Systems*. New York, NY, USA: Association for Computing Machinery. doi:10.1145/3173574.3173905.

Purohit, A. K., Barclay, L., & Holzer, A. (2020). Designing for digital detox: Making social media less addictive with digital nudges. In *Conference on Human Factors in Computing Systems - Proceedings*. Association for Computing Machinery. doi:10.1145/3334480.3382810.

Ren, X., Yu, B., Lu, Y., & Brombacher, A. (2018). Exploring Cooperative Fitness Tracking to Encourage Physical Activity among Office Workers. *Proc. ACM Hum.-Comput. Interact.*,

2. doi:10.1145/3274415. Publisher: Association for Computing Machinery Place: New York, NY, USA.

Rooksby, J., Asadzadeh, P., Rost, M., Morrison, A., & Chalmers, M. (2016). Personal Tracking of Screen Time on Digital Devices. In *Proceedings of the 2016 CHI Conference on Human Factors in Computing Systems*. New York, NY, USA: Association for Computing Machinery. doi:10.1145/2858036.2858055.

Silva, L. M., Ankrah, E. A., Huai, Y., & Epstein, D. A. (2023). Exploring Opportunities for Multimodality and Multiple Devices in Food Journaling. *Proc. ACM Hum.-Comput. Interact.*, *7*. doi:10.1145/3604256.

Silva, L. M., & Epstein, D. A. (2021). Investigating Preferred Food Description Practices in Digital Food Journaling. In *Proceedings of the 2021 ACM Designing Interactive Systems Conference*. New York, NY, USA: Association for Computing Machinery. doi:10.1145/3461778.3462145.

Starke, A. D., Kl\o{}ver\o{}d Brynestad, E. K., Hauge, S., & L\o{}keland, L. S. (2021). Nudging Healthy Choices in Food Search Through List Re-Ranking. In *Adjunct Proceedings of the 29th ACM Conference on User Modeling, Adaptation and Personalization*. New York, NY, USA: Association for Computing Machinery. doi:10.1145/3450614.3464621.

Teuber, M., Leyhr, D., Moll, J., & Sudeck, G. (2022). Nudging digital physical activity breaks for home studying of university students—A randomized controlled trial during the COVID-19 pandemic with daily activity measures. *Frontiers in Sports and Active Living*, *4*. doi:10.3389/fspor.2022.1024996.

Valencic, E., Beckett, E., Collins, C. E., Korousic Seljak, B., & Bucher, T. (2024). Changing the default order of food items in an online grocery store may nudge healthier food choices. *Appetite*, *192*. doi:https://dx.doi.org/10.1016/j.appet.2023.107072.

Valenčič, E., Beckett, E., Collins, C. E., Koroušić Seljak, B., & Bucher, T. (2023). SnackTrack—An App-Based Tool to Assess the Influence of Digital and Physical Environments on Snack Choice. *Nutrients*, *15*. doi:10.3390/nu15020349. Publisher: MDPI.

Vardhan, M., Hegde, N., Merugu, S., Prabhat, S., Nathani, D., Seneviratne, M., Muhammad, N., Reddy, P., Lakshminarasimhan, S., Singh, R., Lorenzana, K., Motwani, E., Talukdar, P., & Raghuveer, A. (2022). Walking with PACE - Personalized and Automated Coaching Engine. In *Proceedings of the 30th ACM Conference on User Modeling, Adaptation and Personalization*. New York, NY, USA: Association for Computing Machinery. doi:10.1145/3503252.3531301 series Title: UMAP '22.

Vigh, E., Attwood, A., & Roudaut, A. (2024). CounterSludge in Alcohol Purchasing on Online Grocery Shopping Platforms. In *Proceedings of the 2024 ACM Designing Interactive Systems Conference*. New York, NY, USA: Association for Computing Machinery. doi:10.1145/3643834.3661595.

Wyse, R., Delaney, T., Stacey, F., Zoetemeyer, R., Lecathelinais, C., Lamont, H., Ball, K., Campbel, K., Rissel, C., Attia, J., Wiggers, J., Yoong, S. L., Oldmeadow, C., Sutherland, R., Nathan, N., Reilly, K., & Wolfenden, L. (2021). Effectiveness of a multistrategy behavioral intervention to increase the nutritional quality of primary school students'web-based canteen lunch orders (click & crunch): Cluster randomized controlled trial. *Journal of Medical Internet Research*, *23*. doi:https://dx.doi.org/10.2196/26054.

Zhang, M. R., Lukoff, K., Rao, R., Baughan, A., & Hiniker, A. (2022). Monitoring Screen Time or Redesigning It? Two Approaches to Supporting Intentional Social Media Use. In *Proceedings of the 2022 CHI Conference on Human Factors in Computing Systems*. New York, NY, USA: Association for Computing Machinery. doi:10.1145/3491102.3517722.
